# Heavy Metal Ion Detection Based on Lateral Flow Assay Technology: Principles and Applications

**DOI:** 10.3390/bios15070438

**Published:** 2025-07-07

**Authors:** Xiaobo Xie, Xinyue Hu, Xin Cao, Qianhui Zhou, Wei Yang, Ranran Yu, Shuaiqi Liu, Huili Hu, Ji Qi, Zhiyang Zhang

**Affiliations:** 1 School of Marine Science and Technology, Harbin Institute of Technology at Weihai, Weihai 264209, China; 15992336124@163.com (X.X.); nhb996888383@163.com (X.H.); lsq18753901011@163.com (H.H.); 2 CAS Key Laboratory of Coastal Environmental Processes and Ecological Remediation, Research Center for Coastal Environmental Engineering and Technology, Yantai Institute of Coastal Zone Research, Chinese Academy of Sciences, Yantai 264003, China; huilihu@hit.edu.cn (X.C.); garytim38@gmail.com (Q.Z.); zhouqianhui066@163.com (W.Y.); yanver@126.com (R.Y.); yrr163163@163.com (S.L.)

**Keywords:** lateral flow assay, heavy metal ion detection, nucleic acids, antigen–antibody, environmental monitoring, food safety

## Abstract

Heavy metal ions pose a significant threat to the environment and human health due to their high toxicity and bioaccumulation. Traditional instrumentations, although sensitive, are often complex, costly, and unsuitable for on-site rapid detection of heavy metal ions. Lateral flow assay technology has emerged as a research hotspot due to its rapid, simple, and cost-effective advantages. This review summarizes the applications of lateral flow assay technology based on nucleic acid molecules and antigen–antibody interactions in heavy metal ion detection, focusing on recognition mechanisms such as DNA probes, nucleic acid enzymes, aptamers, and antigen–antibody binding, as well as signal amplification strategies on lateral flow testing strips. By incorporating these advanced technologies, the sensitivity and specificity of lateral flow assays have been significantly improved, enabling highly sensitive detection of various heavy metal ions, including Hg^2+^, Cd^2+^, Pb^2+^, and Cr^3+^. In the future, the development of lateral flow assay technology for detection of heavy metal ions will focus on multiplex detection, optimization of signal amplification strategies, integration with portable devices, and standardization and commercialization. With continuous technological advancements, lateral flow assay technology will play an increasingly important role in environmental monitoring, food safety, and public health.

## 1. Introduction

Heavy metal ions, characterized by their high density and toxicity, are widely present in natural environments and industrial processes [1]. Due to their non-degradability and bioaccumulation, heavy metal ions pose significant threats to both the environment and human health [2,3]. Heavy metal pollution primarily originates from industrial wastewater, mining activities, agricultural chemical use, and improper disposal of electronic waste [4,5]. These ions enter ecosystems through water, soil, and air, eventually accumulating in organisms through the food chain [6], thereby posing potential risks to human health.

For instance, lead (Pb) is a neurotoxin that causes delayed intellectual development in children and neurological damage in adults [7,8,9]. Cadmium (Cd) exhibits nephrotoxicity and carcinogenicity, leading to osteoporosis via long-term ingestion [10,11,12]. Mercury (Hg), especially methylmercury, crosses the blood–brain barrier, causing irreversible central nervous system damage [13,14]. Hexavalent chromium (Cr(VI)) is highly carcinogenic, linked to lung cancer and skin ulcers [15,16]. Excessive copper (Cu) induces liver damage, while silver (Ag^+^) impairs cellular functions and aquatic ecosystems [17,18]. Arsenic (As), uranium (U), and thallium (Tl) disrupt cellular structures, causing various diseases [19,20,21].

High-concentration heavy metal pollution incidents, such as mercury contamination in the Wanshan Special District of Guizhou (with mercury levels reaching 358.51 mg/kg) [22,23], and heavy metal contamination exceeding safety standards in the Dongjiang River Basin [24], have highlighted the severe threats to human health and ecosystems. These cases underscore the urgent need for better monitoring and remediation, including rapid detection methods like lateral flow assays, to protect human health and the environment. Therefore, the development of rapid and sensitive detection methods, such as lateral flow assays, is crucial for the timely intervention and management of such pollution incidents.

Given the toxicity and widespread presence of heavy metal ions, the development of efficient and sensitive detection methods is crucial for environmental monitoring, food safety, and public health. Traditional detection methods for heavy metal ions include atomic absorption spectroscopy (AAS) [25,26], inductively coupled plasma mass spectrometry (ICP-MS) [27,28], and high-performance liquid chromatography (HPLC) [29,30]. While these methods offer high sensitivity and accuracy, they typically require complex sample preparation, expensive instrumentation, and specialized technical expertise, making them unsuitable for on-site rapid detection. Consequently, the development of a rapid, simple, and cost-effective heavy metal ion detection technology has become a research focus.

In addition to traditional instrumental analysis, electrochemical methods (such as voltammetry [31,32,33], which detects heavy metal ions by measuring current changes from redox reactions on electrode surfaces, with a detection limit of 0.1 nM) and spectroscopic techniques (such as surface enhanced Raman scattering (SERS) spectroscopy, which quantifies ions through characteristic Raman signals generated by interactions between heavy metal ions and nanomaterials [34,35], achieving a detection limit as low as 1 pM) are also used for heavy metal ion detection. However, these technologies face significant limitations: voltammetry is susceptible to matrix interference and requires frequent electrode maintenance, while SERS relies on expensive spectrometers and professional data analysis, making it difficult to deploy on-site and unsuitable for real-time screening in sudden pollution incidents.

Lateral flow assay (LFA) is more suitable for on-site rapid detection due to its minimal operation (visual or smartphone reading within 10–30 min after sample addition), low cost (each test strip < $1), strong portability (no power supply or instruments required), and excellent resistance to matrix interference. By integrating nanomaterial labeling and signal amplification strategies, LFA can enhance sensitivity to the pM level and is compatible with various complex samples. In contrast, other technologies often rely on specialized instruments or complicated pretreatment, making them difficult to popularize in emergency monitoring and other on-site scenarios. Its core significance lies in enabling real-time on-site sampling and analysis of water, soil, and other samples during sudden heavy metal pollution incidents, providing immediate data support for pollution source control and early warning of population exposure risks-thereby filling the gap of traditional technologies in scenarios requiring ‘rapid response and on-site screening’.

In recent years, emerging rapid detection technologies for heavy metal ions have focused on integrating nanomaterials, microfluidics, and optoelectronic devices. For example, surface-enhanced Raman spectroscopy (SERS) combined with microfluidic chips enables ultrasensitive detection by amplifying characteristic spectral signals [35], while electrochemical sensors based on metal–organic frameworks (MOFs) achieve sub-nanomolar sensitivity through in situ redox reactions [36]. Fluorescent nanoprobes (e.g., quantum dots) have also been developed for real-time imaging of ions in biological samples [37], and smartphone-integrated detection systems are being designed to realize portable [38], high-throughput analysis. These technologies highlight trends toward miniaturization, high sensitivity, and intelligence in on-site rapid detection, though challenges remain in cost reduction and anti-interference capability for practical applications.

Lateral flow assay (LFA) technology is a rapid detection method known for its simplicity, low cost, and quick response, widely used in the detection of biomarkers, pathogens, and environmental pollutants [39,40,41,42,43]. A typical lateral flow test strip consists of a sample pad, conjugate pad, nitrocellulose membrane (NC membrane), and absorbent pad. When a sample solution is applied to the sample pad, the target analyte in the sample binds to the labeled conjugate on the conjugate pad, forming a complex. This complex then migrates along the NC membrane via capillary action, binding to the capture reagent immobilized on the membrane to form a visible test line [44]. The advantages of LFA technology lie in its independence from complex instrumentation and specialized technical personnel, making it suitable for on-site rapid detection [45,46,47].

In recent years, advancements in nanomaterials and biosensing technologies have significantly enhanced the application of LFA technology in heavy metal ion detection. Although plenty of work has been performed to develop rapid detection methods using LFA technology [48,49,50,51,52], there is no review specially focusing on the development of LFA-based heavy metal ion detection techniques. This review summarizes recent developments in heavy metal ion detection methods based on LFA technology. It focuses on the detection principles, detection limits, as well as signal readout methods and the types of detection samples for various heavy metal ions. By systematically analyzing existing research, this review aims to provide a reference for the future development of more efficient and sensitive heavy metal ion detection technologies.

## 2. Detection Strategies for Heavy Metal Ions Based on LFA Technology

**Figure 1:**
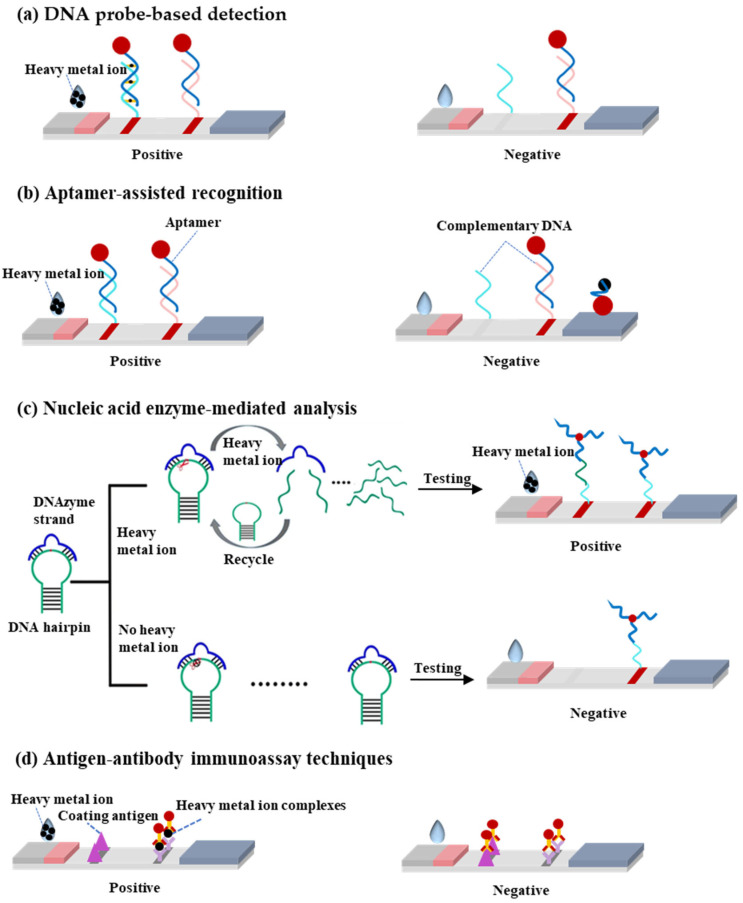
(**a**) Schematic diagram of DNA probe-based detection principle. (**b**) Schematic diagram of aptamer-assisted recognition principle. (**c**) Schematic diagram of nucleic acid enzyme-mediated analysis principle. (**d**) Schematic diagram of antigen–antibody immunoassay technique principle.

This review systematically examines heavy metal ion detection methods utilizing LFA technology, with a focused exploration of four principal detection strategies and their recent advancements. From perspective of sensing principle, the LFA-based heavy metal detection methods mainly include four types: (1) DNA probe-based detection, (2) aptamer-assisted recognition, (3) nucleic acid enzyme-mediated analysis, and (4) antigen–antibody immunoassay techniques (Figure 1). In the following paragraphs, we will discuss the detailed sensing principles and applications for various meatal ions.

### 2.1. DNA Probe-Based LFA Technology

The DNA probe-based lateral flow assay represents a rapid and portable detection technology for heavy metal ions, with its core principle relying on the specific recognition capability of DNA molecules toward target metal ions. The detection mechanism involves three key steps (Figure 1a):

(i) Specific binding: DNA probes (e.g., T-rich sequences) form stable complexes or chemical bonds with target ions (e.g., Hg^2+^ through T-Hg^2+^-T coordination).
(ii) Signal labeling: Gold nanoparticles (AuNPs) are labeled by a thiol terminated probing DNA sequence, which contains the recognition bases or groups for target metal ions.
(iii) Visual readout: The labeled gold nanoparticle will be captured by the complementary DNA probes at the T line by forming stale double DNA structures, producing distinct red bands whose intensity correlates with target concentration.

For the DNA probe-based detection principle, it has advantages of simple probe design and high selectivity. To date, this strategy has been used for detection of various heavy metal ions, including Cu^2+^, Hg^2+^, Ag^+^, Pb^2+^, As^3+^, etc. By rational experimental design, these methods exhibit satisfactory sensitivity, and the detection limit can reach as low as nM levels. More importantly, these methods also are applicable to different real samples including environmental samples, food samples, and human fluids, demonstrating its applicability for on-site environmental monitoring and point-of-care testing applications. Details are provided in Table 1.

Abrams et al. [53] utilized gold nanoparticles (AuNPs) as signal labels and employed azide-DNA and alkyne/biotin-DNA as recognition units. Through a click chemistry reaction, the linkage product was formed and conjugated with AuNPs to create a complex, enabling the visual detection of Cu^2+^ with a detection limit of 100 nM. Similarly, Wang et al. [54] employed AuNPs and modified single-stranded DNA (ssDNA) to detect Cu^2+^ via click chemistry, achieving detection limits of 5 nM (visual) and 4.2 nM (quantitative). This method is characterized by its simplicity, low cost, and high sensitivity, making it suitable for on-site diagnostics and environmental monitoring (e.g., detection of Cu^2+^ contamination in drinking water or industrial wastewater).

**Figure 2:**
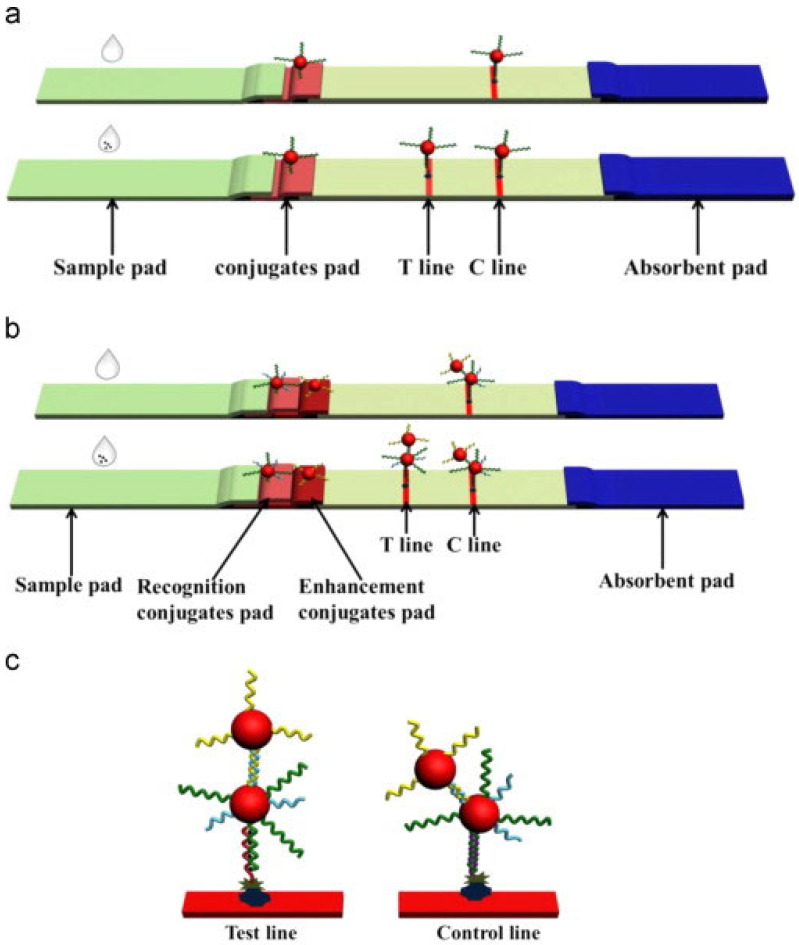
(**a**) The sensing principle of the traditional lateral flow strip. (**b**) The sensing principle of the signal amplified lateral flow strip. (**c**) The test line and control line of the signal amplified lateral flow strip. Adapted with permission from [55].

Zhu et al. [55] developed a lateral flow strip based on AuNPs and ssDNA probes for the rapid and ultrasensitive detection of Hg^2+^ in water. This method relies on the specific binding of T-rich ssDNA probes to Hg^2+^, forming T-Hg^2+^-T mismatch structures. AuNPs were used as signal labels, and signal amplification was achieved through enhanced probe hybridization, as illustrated in Figure 2. This approach enables simultaneous detection and signal amplification in a single step, with a visual detection limit of 25 pM and a quantitative detection limit of 7.5 pM. The method has been successfully applied to real samples such as tap water, tea water, and lake water, demonstrating its broad application potential.

Liu et al. [56], Guo et al. [57], Cheng et al. [58], and Yao et al. [59] developed various highly sensitive Hg^2+^ detection methods based on the T-Hg^2+^-T specific recognition mechanism, combined with AuNPs and DNA probes. These methods achieved Hg^2+^ detection through signal amplification techniques such as isothermal nucleic acid amplification and surface-enhanced Raman spectroscopy (SERS), with detection limits ranging from 0.36 pM to 5 nM.

**Figure 3:**
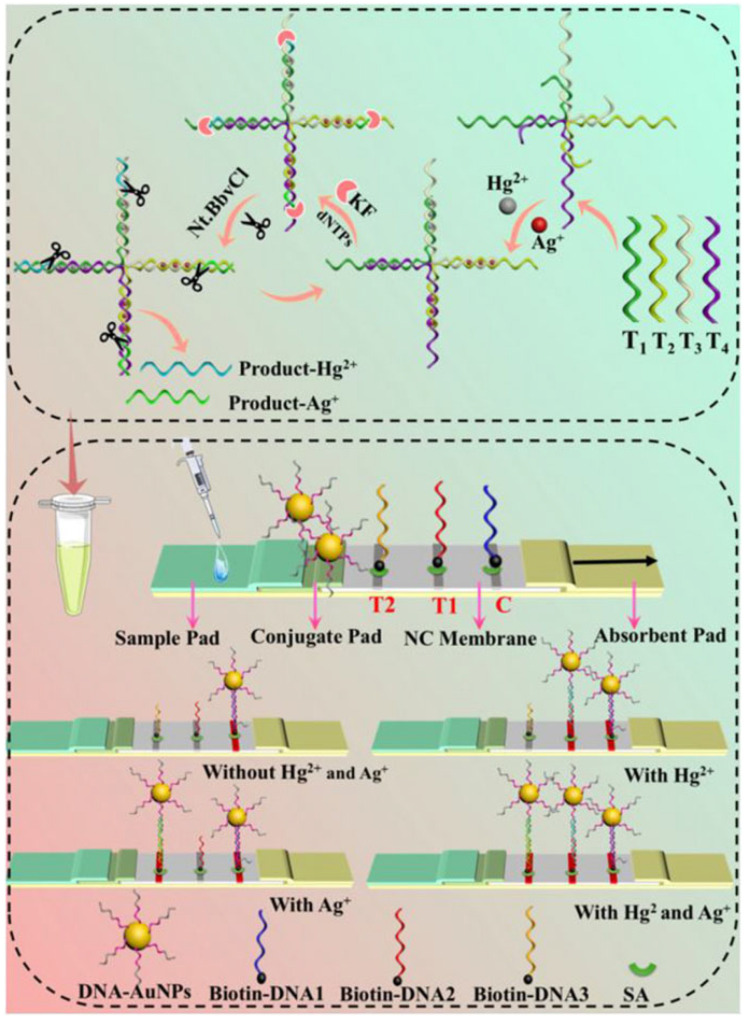
Schematic diagram of LFA based on an isothermal amplification strategy for the detection of Hg^2+^ and Ag^+^. Adapted with permission from [60].

Wang et al. [60] utilized the T-Hg^2+^-T and C-Ag^+^-C structures for specific recognition and employed isothermal nucleic acid amplification for signal amplification to detect Hg^2+^ and Ag^+^ in water samples. The detection limits were 2.19 pM for Hg^2+^ and 5.41 pM for Ag^+^, as illustrated in Figure 3. This method exhibits high sensitivity and selectivity, making it suitable for on-site rapid detection.

**Figure 4:**
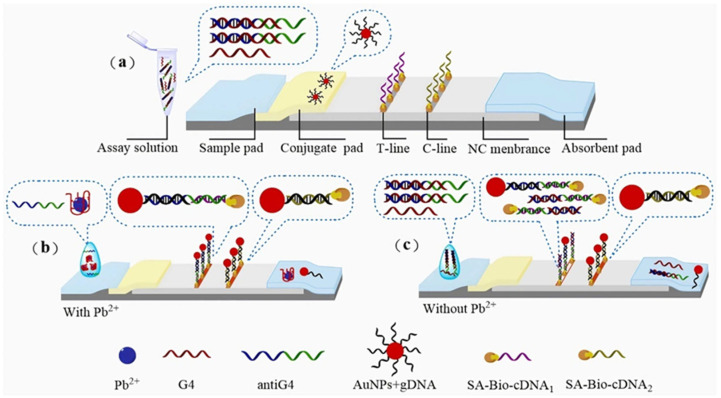
Principle of test strip detection: (**a**) structural diagram; (**b**) with Pb^2+^; (**c**) without Pb^2+^. Adapted with permission from [61].

Ren et al. [61] employed AuNPs and G-quadruplex structures to detect Pb^2+^ in bottled drinking water. The method relies on lead ion-induced G-quadruplex formation and signal amplification through the release of anti-G4 single-stranded DNA. The detection limits were 20 nM (visual) and 7.32 nM (quantitative), as shown in Figure 4. Similarly, Wang et al. [62] developed a Pb^2+^ detection sensor based on the G-quadruplex structural switching mechanism, achieving a detection limit of 25 nM. This method combines the high specificity of G-quadruplex structures with the simplicity of lateral flow test strips, making it suitable for on-site rapid detection.

Zhang et al. [63] utilized a short single-stranded DNA (Apt-21) that binds to As(III) to form an As(III)-Apt-21 complex, serving as the recognition unit for As(III). Signal amplification was achieved through As(III)-induced aggregation of AuNPs, enabling the detection of As(III) in tap water and river water with a detection limit of 2.4 nM. This method combines the high specificity of short-chain DNA with the colorimetric properties of gold nanoparticles, making it suitable for on-site rapid detection.

This part of the research focuses on using DNA probes to detect heavy metal ions. The core principle is to form stable complexes through the specific binding of specific DNA sequences to target ions. Although the research cases vary, they all demonstrate the technology’s ability to detect multiple ions. This technology is generally easy to operate, low-cost, and highly sensitive, showing great potential in fields such as on-site diagnosis and environmental monitoring. However, this strategy can be only used for some special heavy metal ions such as Cu^2+^, Hg^2+^, Ag^+^, Pb^2+^, As^3+^, etc. In addition, the design and synthesis of some DNA probes with chemical modification are still complex, such as the modification of azide- and alkyne for the Cu^2+^ DNA probe.

Common challenges: Despite its advantages, this technology faces several limitations: (i) chemical modification of DNA probes (e.g., azide/alkyne for Cu^2+^ detection) increases synthesis complexity and cost; (ii) detection is primarily limited to specific ions (Hg^2+^, Pb^2+^) due to reliance on sequence-specific coordination; (iii) matrix interference from coexisting ions (e.g., Na^+^ in environmental water) may reduce binding specificity. These challenges highlight the need for universal probe design and anti-interference optimization.

### 2.2. Aptamer-Based LFA Technology

The aptamer-based lateral flow assay represents a cutting-edge biosensing platform that employs artificially selected nucleic acid aptamers for specific target detection. This innovative technology operates through three fundamental mechanistic steps:

(i) Specific binding: Aptamers bind to target heavy metal ions (e.g., Hg^2+^, Cd^2+^) with high affinity through their unique three-dimensional structures, inducing significant conformational changes.
(ii) Signal labeling: The spatial distribution or aggregation state of reporter molecules (e.g., gold nanoparticles, fluorescent dyes) conjugated to aptamers is altered by these conformational changes.
(iii) Visual readout: Signal molecules accumulate at the test line (T-line), producing colored bands whose intensity correlates with target concentration, while the control line (C-line) validates assay performance.

The combination of molecular recognition precision and lateral flow simplicity positions this technology as a powerful tool for point-of-care testing and field-deployable analysis. Continuous improvements in aptamer selection and signal enhancement methodologies promise to further expand its application scope and detection capabilities. Details are provided in Table 2.

In the field of environmental monitoring, aptamer technology has demonstrated significant value for heavy metal ion detection, as detailed in Table 3. Specific examples include the following.

**Figure 5:**
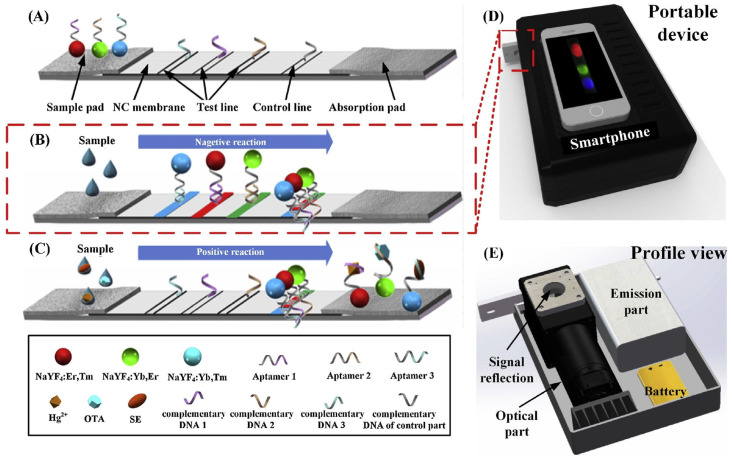
Schematic illustration of LFAA for simultaneous multiple targets detection. (**A**) The structure of the developed LFAA. (**B**) In the absence of target, UCNP probes were separately hybridized with the corresponding complementary DNA. (**C**) In the presence of targets, the aptamers preferentially bonded to the corresponding targets and caused fewer aptamers to be hybridized with complementary DNA, thereby liberating UCNPs and resulting in fluorescence decrease. (**D**) A smartphone-based portable device is used to read the detection results. (**E**) The schematic of the smartphone-based portable device. Adapted with permission from [64].

Jin et al. [64] utilized the fluorescence emission properties of upconversion nanoparticles (UCNPs) under near-infrared light excitation. By leveraging the specific binding of aptamers to target analytes, they achieved a highly sensitive and selective detection of Hg^2+^ with a detection limit of 25 nM. This detection platform completed the analysis of real water samples (e.g., tap water) within 30 min, demonstrating its broad application potential in environmental monitoring and food safety, as illustrated in Figure 5.

**Figure 6:**
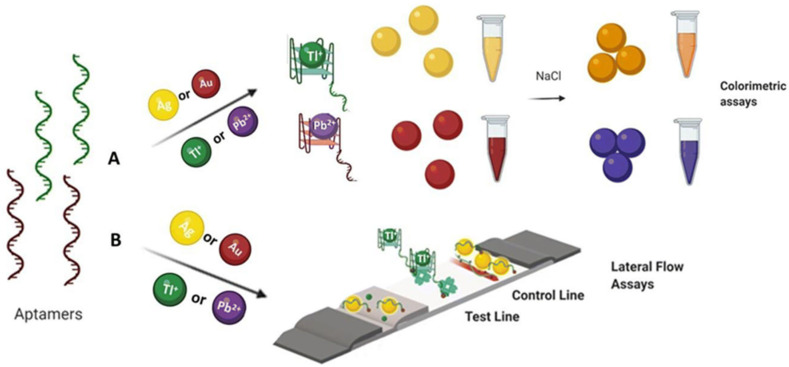
Schematic illustration of (**A**) colorimetric and (**B**) lateral flow assay approaches for the detection of Tl(I) and Pb(II) ions. Adapted with permission from [67].

Irfan et al. [65] and Wu et al. [66] developed lateral flow test strips based on aptamers and fluorescence technology for the detection of Cd^2+^ and Hg^2+^ in river water, achieving detection limits of 30 nM and 0.65 nM, respectively. Srinivasan et al. [67] utilized the specific binding of aptamers to Tl^+^ to enable rapid detection of thallium ions, as shown in Figure 6.

Berlina et al. [68] designed a lateral flow test strip based on phenylboronic acid and oligocytosine aptamers for the highly selective detection of Pb^2+^, with an instrumental detection limit of 0.24 nM and a visual detection limit of 4.8 nM, completing the detection within 5 min. In summary, aptamer-based LFA technology offers the advantages of simplicity, low cost, and high sensitivity.

The key to this technology is the high-affinity binding between aptamers and target heavy metal ions, achieving specific recognition. Research has been conducted on the detection of various ions such as Hg^2+^, Cd^2+^, Tl^+^, and Pb^2+^. Its advantages include simple operation, low cost, and high sensitivity, making it highly valuable in environmental monitoring. However, the aptamer screening process is complex and time-consuming, and the binding stability between aptamers and ions may be affected by other components in the sample, posing certain limitations in the detection of complex samples in practice.

Common challenges: The technology is constrained by (i) time-consuming aptamer screening (SELEX process requiring weeks to months); (ii) conformational instability of aptamers under varying salt/temperature conditions, leading to inconsistent detection signals; (iii) limited multiplexing capability for simultaneous ion analysis.

### 2.3. Nucleic Acid Enzyme-Based LFA Technology

The nucleic acid enzyme-based lateral flow assay is an advanced detection platform that leverages the specific catalytic activity of DNAzymes or MNAzymes for rapid heavy metal ion analysis. The detection mechanism operates through three precisely coordinated steps:

(i) Specific binding: Target heavy metal ions (e.g., Pb^2+^) specifically activate nucleic acid enzymes (e.g., 8–17 DNAzyme), initiating cleavage of substrate DNA strands.
(ii) Signal labeling: The cleavage reaction releases labeled fragments (e.g., fluorophore-conjugated or gold nanoparticle-tagged DNA segments).
(iii) Visual readout: Released markers are captured at the T-line, generating visible signals (e.g., red bands) with intensity proportional to ion concentration.

This innovative approach combines the precision of nucleic acid enzymes with the simplicity of lateral flow technology, making it particularly valuable for environmental monitoring and field-deployable diagnostics where both accuracy and speed are essential. The system’s modular design also permits adaptation for various metal ion targets through appropriate enzyme selection. Details are provided in Table 3.

**Figure 7:**
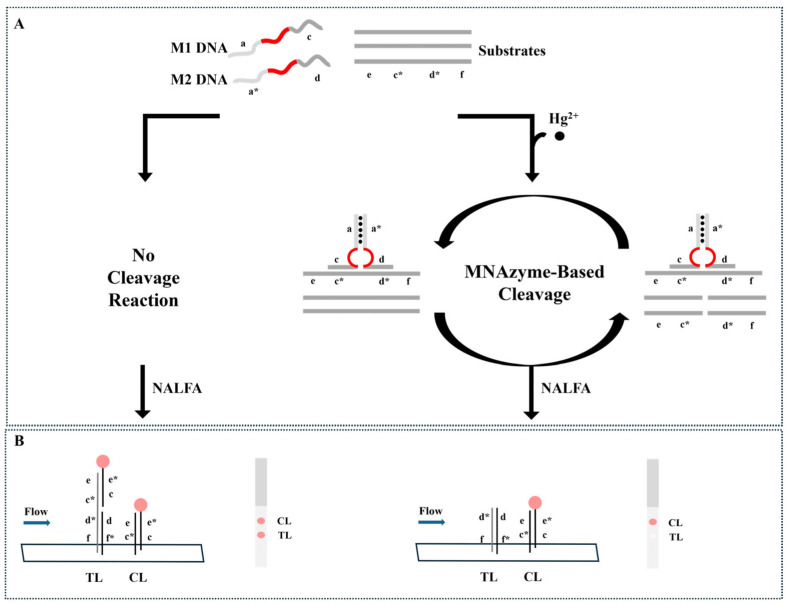
Schematic illustration of the Hg^2+^ detection assay where (**A**) MNAzyme-based cleavage reaction occurs only in the presence of Hg^2+^ and (**B**) the reaction products are analyzed by the NALFA strip. Adapted with permission from [69].

Kim et al. [69] developed a colorimetric detection method based on multicomponent nucleic acid enzymes (MNAzymes) and lateral flow test strips for the rapid detection of Hg^2+^ in tap water. This method utilizes MNAzymes containing T-T mismatches to specifically recognize Hg^2+^ and activate catalytic activity, leading to substrate cleavage. The cleaved substrate fails to produce a color signal on the test strip, while the uncleaved substrate generates a distinct red signal, enabling signal amplification. The detection limit of this method is 9.34 nM, as illustrated in Figure 7.

**Figure 8:**
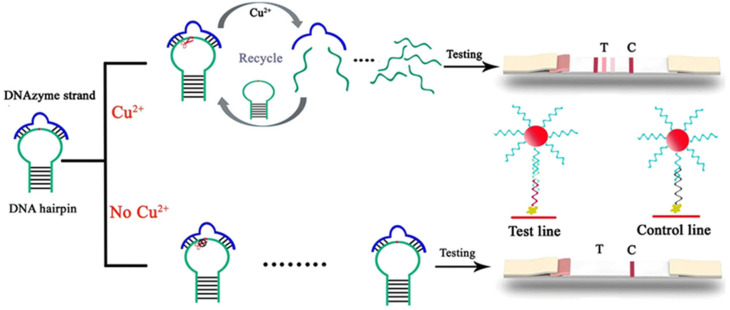
Schematic representation of the catalytic and stem-loop signal amplification strategy-based Cu^2+^ lateral flow assay. Adapted with permission from [70].

Wang et al. [70] developed a lateral flow assay strip based on DNAzyme catalysis and stem-loop structure signal amplification. This innovative approach utilizes Cu^2+^ to activate DNAzyme, which cleaves the stem-loop DNA substrate to release ssDNA fragments. The liberated DNAzyme can subsequently bind to new stem-loop DNA molecules, enabling multiple turnover cycles where each DNAzyme molecule catalyzes the production of numerous ssDNA fragments. This amplification strategy achieves highly sensitive and specific detection of Cu^2+^ with a remarkable detection limit of 31.5 nM, as illustrated in Figure 8. The approach offers high selectivity and the advantage of not requiring complex instrumentation.

Mazumdar et al. [71], Wang et al. [72], and Pei et al. [73] employed DNAzyme for the detection of Pb^2+^ in environmental samples, achieving detection limits of 5 μM, 20 nM, and 0.05 nM, respectively. Additionally, Fang et al. [74] employed DNAzyme to specifically recognize Cu^2+^ with a detection limit of 10 nM.

This technology detects heavy metal ions by amplifying the detection signal through enzyme-catalyzed reactions that produce colored or fluorescent products. Many studies have been carried out on various ions such as Hg^2+^, Cu^2+^, and Pb^2+^. Its advantages include high selectivity and the absence of the need for complex equipment, making it suitable for on-site rapid detection. However, it also has obvious disadvantages. The activity of the enzyme is easily affected by environmental factors such as temperature and pH, which may lead to unstable test results. Moreover, the storage conditions for enzymes are harsh, increasing the cost and difficulty of use.

Common challenges: Key limitations include (i) enzyme activity susceptibility to environmental factors (e.g., pH and temperature fluctuations); (ii) high cost and harsh storage requirements (4 °C or below) for nucleic acid enzymes; (iii) potential false positives from non-specific cleavage in complex matrices.

### 2.4. Antigen–Antibody-Based LFA Technology

The antibody–antigen-based lateral flow immunoassay represents a well-established immunochromatographic detection methodology, whose operational mechanism comprises three critical phases:

(i) Specific binding: Immobilized antibodies (or antigens) on the test strip specifically bind to target analytes (e.g., metal ion–carrier protein complexes) in samples.
(ii) Signal labeling: Gold-conjugated antibodies form immunocomplexes that migrate via capillary action.
(iii) Visual readout: Immunocomplexes accumulate at the T-line, producing colored bands, while the C-line confirms assay validity.

Heavy metal ions, due to their small molecular weight and lack of immunogenicity, are classified as haptens and cannot directly stimulate antibody production in the immune system. Therefore, they must be chemically conjugated to carrier proteins (such as BSA, KLH, OVA, etc.) to form complete antigens [75], which can then be used to immunize animals for antibody production.

The combination of robust immunological specificity with simple visual readout has established this platform as a gold standard for rapid diagnostic applications across multiple sectors. Continuous improvements in bioreceptor engineering and detection modalities promise to further expand their utility and performance characteristics. Details are provided in Table 4.

**Figure 9:**
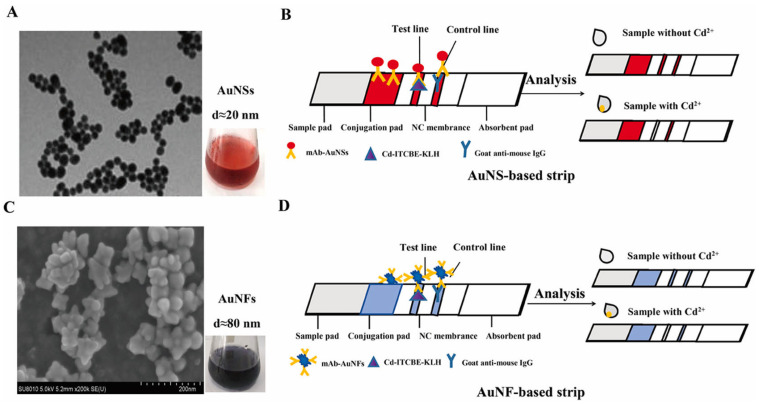
Characterization and Detection Principle of Cd^2+^ Using AuNS and AuNF-Labeled Lateral Flow Test Strips. (**A**) Transmission electron microscopy (TEM) image and colloidal solution of AuNS. (**B**) Schematic diagram of the detection principle for Cd^2+^ using AuNS-based lateral flow test strips. (**C**) Scanning electron microscopy (SEM) image and colloidal solution of AuNF. (**D**) Schematic diagram of the detection principle for Cd^2+^ using AuNF-based lateral flow test strips. Adapted with permission from [76].

For example, Ling et al. [76] used lateral flow test strips to detect Cd^2+^ in environmental water samples. By leveraging the specific binding of gold nanospheres (AuNS) and gold nanoflowers (AuNF)-labeled antibodies to target Cd^2+^, immune complexes were formed and captured at the test line, producing visible red bands. This enabled highly sensitive detection of Cd^2+^, with visual detection limits of 3.34 nM (AuNS) and 0.27 nM (AuNF), as illustrated in Figure 9. This method offers high selectivity and does not require complex instrumentation, making it suitable for on-site rapid detection.

**Figure 10:**
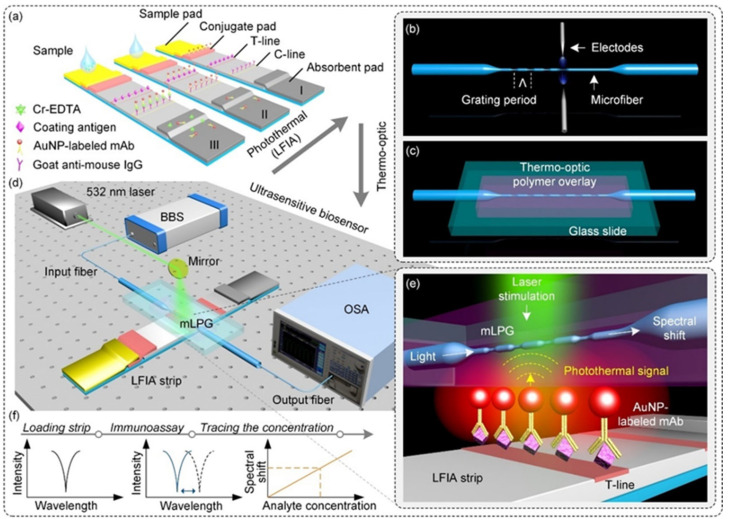
(**a**) Illustration of the competitive lateral flow immunoassay. (**b**) Schematic of the microfiber long-period grating (mLPG) and its fabrication procedure using the electric arc discharge method. (**c**) Schematic of the encapsulated mLPG with a UV-curable polymer overlay supported by a glass slide. (**d**) Illustration of the experimental setup for the proposed biosensor. (**e**) Sensing mechanism of the signal transduction. (**f**) Spectral change during measurement with the proposed biosensor. Adapted with permission from [77].

Huang et al. [77] developed a photothermal lateral flow immunoassay (LFIA) sensor using micro-fiber long-period grating (mLPG) and AuNPs for the detection of chromium ions (Cr^3+^) in water and serum samples. The sensor leverages the localized surface plasmon resonance (LSPR) effect of AuNPs to generate photothermal signals. Combined with the thermo-optic conversion of a polymer encapsulation layer, the concentration information of the target analyte is converted into spectral changes in the mLPG. Through the signal amplification of the photothermal effect, the sensor achieved highly sensitive detection of Cr^3+^ with a detection limit of 4.21 nM, as illustrated in Figure 10.

In recent years, lateral flow immunoassay technology based on nanomaterials (e.g., gold and silver nanoparticles) and monoclonal antibodies has been widely applied for the detection of heavy metal ions such as Cr^3+^, UO_2_^2+^, Hg^2+^, Cd^2+^, and Pb^2+^ [78,79,80,81,82,83,84,85,86]. By combining competitive or non-competitive immunoassays with signal amplification strategies (e.g., surface-enhanced Raman spectroscopy (SERS), fluorescent microspheres), this technology has achieved high sensitivity (detection limits ranging from pM to μM levels), rapid response (3–4 min), and portable detection. These advancements make LFIA suitable for on-site rapid screening of heavy metal ions in complex matrices such as environmental water samples and food.

This technology detects heavy metal ions by forming immune complexes through the specific binding of antigens and antibodies. Research has been carried out on various ions such as Cd^2+^, Cr^3+^, U^4+^, Hg^2+^, and Pb^2+^.

Common challenges: This approach is hindered by (i) high production costs for monoclonal antibodies (typically $1000+/mg); (ii) weak immunogenicity of metal ions, requiring unstable conjugation with carrier proteins; (iii) cross-reactivity risks with similar metal-chelate complexes.

## 3. Conclusions and Future Perspectives

Lateral flow assay technology has emerged as a transformative platform for rapid, on-site detection of heavy metal ions, addressing critical needs in environmental monitoring, food safety, and public health. This review summarized four principal LFA methodologies, each employing distinct recognition mechanisms and using different signal readout methods for various detection samples to achieve sensitive and specific detection of heavy metal ions.

The DNA probe-based approach capitalizes on the programmable binding between synthetic oligonucleotides and target ions, exemplified by T-Hg^2+^-T coordination and G-quadruplex formation with Pb^2+^. These systems achieve exceptional sensitivity (e.g., 0.0015 ppb for Hg^2+^) through gold nanoparticle labeling, though they face challenges in probe design complexity and matrix interference. The nucleic acid enzyme-based method utilizes the specific catalytic activation of nucleic acid enzymes (such as DNAzyme and MNAzyme) by targeting heavy metal ions (such as Pb^2+^ and Hg^2+^), cleaving the substrate DNA strand to release labeled fragments and generate detection signals (such as red bands), thus achieving high-sensitivity detection (for example, the detection limit of Pb^2+^ can reach 0.05 nM). However, the activity of the enzyme is easily affected by the environment and the storage conditions are harsh.

Aptamer-based platforms employ synthetic nucleic acid receptors that undergo conformational changes upon ion binding, enabling multiplexed detection of Hg^2+^, Cd^2+^, and Tl^+^ through innovative signal reporters. While offering superior adaptability, these systems require labor-intensive aptamer development. The well-established antigen–antibody format provides robust detection of metal-chelate complexes (e.g., Cd^2+^-EDTA) with commercial-ready test strips, though at higher antibody production costs.

Comparative analysis reveals technology-specific advantages: DNA probes deliver ultra-sensitive and cost-effective detection, nucleic acid enzymes enable isothermal amplification, aptamers permit customizable multiplexing, while immunoassays offer regulatory-friendly solutions. Emerging innovations in nanomaterial labels, smartphone-based quantification, and integrated multi-analyte strips are addressing current limitations in sensitivity, cost, and field applicability. Standardization of these advanced LFA systems will be crucial for widespread adoption in global monitoring programs, particularly for addressing acute heavy metal contamination events and ensuring food chain safety. The continuous evolution of LFA technologies promises to reshape environmental and health surveillance paradigms through decentralized, user-friendly metal ion detection, as shown in Table 5 specifically.

In the future, the development of lateral flow analysis technology will focus on the following directions, with clarified application scenarios and retained references:

(i) Multiplex Detection

Scenario: Industrial wastewater treatment plants can utilize multiplex LFIA strips to simultaneously monitor Pb^2+^, Cd^2+^, and Hg^2+^, enabling real-time adjustment of purification processes. This addresses the complex metal contamination challenges observed in the Dongjiang River Basin.

(ii) Signal Amplification Optimization

Scenario: During drinking water source emergencies, LFIA integrated with nanomaterial-based amplification can achieve ultrasensitive Hg^2+^ detection to prevent toxic substance infiltration. The electrochemical signal enhancement strategies discussed further optimize sensitivity for field applications.

(iii) Integration with Portable Devices

Scenario: Fishery aquaculture monitoring can employ smartphone-integrated LFIA systems to quantify Cu^2+^ in pond water within 10 min, building on the portable detection frameworks developed for tap water analysis. IoT technology enables real-time data transmission to prevent metal accumulation in seafood.

(iv) Standardization and Commercialization

Scenario: Grassroots public health centers can adopt standardized LFIA kits for rapid screening of heavy metal exposure in children, leveraging the cost-effective design suitable for poor regions.

(v) SERS-Microdevice Integration

Recent advancements in SERS-based microdevices [34,78,87] show promise for LFA integration. As demonstrated by Lee et al. [88], a SERS-LFA hybrid device with microfluidic pretreatment achieves sub-pM Hg^2+^ detection in environmental samples, combining on-chip nucleic acid extraction with multiplexed SERS amplification. This integration reduces sample volume to 10 μL and analysis time to 20 min, enabling point-of-care testing for sudden heavy metal pollution incidents.

With continuous technological advancements, lateral flow analysis is expected to play an increasingly important role in heavy metal ion detection, addressing critical challenges in environmental protection, food safety, and public health.

## Figures and Tables

**Table 1:** Detection of Heavy Metal Ions Using DNA Probe-Based LFA Technology.

Metal Type	Material	Identification Unit	Signal Readout	Detection Limit	Detection Samples	Reference
Cu^2+^	AuNPs	Azide-DNA and alkyne/biotin-DNA	Colorimetric/Reading device	100 nM	Tap water and human serum	[53]
Cu^2+^	AuNPs	Azide- and alkyne-modified ssDNA	Colorimetric/Reading device	5 nM	Municipal water and river water	[54]
Hg^2+^	AuNPs	ssDNA	Colorimetric/Image J	25 pM	Tap water, tea water, and lake water	[55]
Hg^2+^	AuNPs	T-Hg^2+^-T	Colorimetric/Reading device	5 nM	River water	[56]
Hg^2+^	AuNPs	T-Hg^2+^-T	Colorimetric/biosensor	2.53 nM	Hg(II)-containing aqueous solution	[57]
Hg^2+^	AuNPs	T-Hg^2+^-T	Colorimetric/Mobile analysis	4 nM	Tap water	[58]
Hg^2+^	Au@AgNPs	T-Hg^2+^-T	SERS/LabRAM HR Evolution system	0.36 pM	Tea	[59]
Hg^2+^ Ag^+^	AuNPs	T-Hg^2+^-T and C-Ag^+^-C	Colorimetric/ImageJ	Hg^2+^: 2.19 pM Ag^+^: 5.41 pM	River water and Tap water	[60]
Pb^2+^	AuNPs	antiG4 single-stranded DNA	Colorimetric/Image J	20 nM	Drinking water	[61]
Pb^2+^	AuNPs	Domain 2 (G-rich sequence) in DNA 1-2 probe	Colorimetric/Colorimetric card	25 nM	Solutions containing different concentrations of lead ions	[62]
As^3+^	AuNPs	As^3+^-Apt-21 complex	Colorimetric	2.4 nM	Tap water and River water	[63]

**Table 2:** Detection of Heavy Metal Ions Using Aptamer-Based LFA Technology.

Metal Type	Material	Identification Unit	Signal Readout	Detection Limit	Detection Samples	Reference
Hg^2+^	Upconversion nanoparticles (UCNPs)	Aptamer	Fluorescence/ImageJ	25 nM	Tap water	[64]
Cd^2+^	30 nt DNA probe	Cy5-labeled aptamer	Fluorescence	30 nM	River water	[65]
Hg^2+^	AuNPs	Specific oligonucleotide probe	Fluorescence/Fluorescence reader	0.65 nM	River water	[66]
Tl^+^	AuNPs AgNPs	Aptamer	Colorimetric/ImageJ	AuNPs: 7.4 µM, AgNPs: 6.3 µM	Distilled water, River water, Human serum	[67]
Pb^2+^	AuNPs	Phenylboronic acid and oligocytosine	Colorimetric/TotalLab TL120	4.8 nM	Drinking water	[68]

**Table 3:** Detection of Heavy Metal Ions Using Nucleic Acid Enzyme-Based LFA Technology.

Metal Type	Material	Identification Unit	Signal Readout	Detection Limit	Detection Samples	Reference
Hg^2+^	AuNPs	MNAzyme	Colorimetric/ImageJ	9.34 nM	Tap water	[69]
Cu^2+^	AuNPs	DNAzyme	Colorimetric	31.5 nM	Tap water and river water	[70]
Pb^2+^	AuNPs	8–17 DNAzyme	Colorimetric	5 μM	Paint	[71]
Pb^2+^	AuNPs	17E DNAzyme	Colorimetric	20 nM	Tap water, river water, and pool water	[72]
Pb^2+^	AuNPs	8–17 DNAzyme	Colorimetric	0.05 nM	Drinking water	[73]
Cu^2+^	AuNPs	DNAzyme	Colorimetric/Strip reader	10 nM	Aqueous solution	[74]

**Table 4:** Detection of Heavy Metal Ions Using Antigen–Antibody-Based LFA Technology.

Metal Type	Material	Identification Unit	Signal Readout	Detection Limit	Detection Samples	Reference
Cd^2+^	AuNS AuNF	Monoclonal antibody (MAb)	Colorimetric/Reading device	AuNS: 3.34 nM, AuNF: 0.27 nM	Drinking water, tap water and laboratory deionized water	[76]
Cr^3+^	AuNPs	Monoclonal antibody (MAb)	measure the spectral shift in microfiber long-period gratings	4.21 nM	solutions containing different concentrations of Cr(III) ions	[77]
Cr^3+^	AgNPs	Anti-Cr^3+^-EDTA monoclonal antibody	SERS	0.192 pM	Distilled water, tap water, and environmental water samples	[78]
U^4+^	AuNPs	Monoclonal antibody (12F6)	Colorimetric/Image J	6 nM	Groundwater	[79]
Cr^3+^ Cr^6+^	AuNPs	Monoclonal antibody (McAb)	Colorimetric/Reading device	0.962 μM	Water samples from the Pearl River and West Lake	[80]
Pb^2+^	AuNPs	Anti-Pb-DTPA monoclonal antibody	Colorimetric	0.241 μM	Water samples from the Pearl River	[81]
Cd^2+^	AuNPs	Anti-Cd(II)-ITCBE monoclonal antibody (3A9)	Colorimetric/Scanning reading device	1.78 nM	Tap water	[82]
Cd^2+^	FMs	Monoclonal antibody (mAb 2F7)	Colorimetric and fluorescence/Reading device	17.17 nM	Rice	[83]
Cd^2+^	AuNPs LMs PDA	Anti-cadmium monoclonal antibody (4A9)	Colorimetric/Reading device	44.48 nM, 0.89 nM, 0.89 nM	Asparagus	[84]
Cd^2+^	AuNPs	Monoclonal antibody (2A81G5)	Colorimetric/Reading device	0.89 nM	Tap water	[85]
Hg^2+^ Cd^2+^ Pb^2+^	AuNPs	Monoclonal antibody	Colorimetric/Epson 3200 Photo Scanner	8 nM, 6 nM, and 6 nM	Mineral water, tap water, and lake water	[86]

**Table 5:** Performance and Advantages Comparison of Four Heavy Metal Ion Detection Strategies.

Detection Strategy	Recognition Mechanism	Core Advantages
DNA Probe-Based LFA Technology	Specific binding of DNA sequences (e.g., T-rich, G-quadruplex) to metal ions (e.g., T-Hg^2+^-T coordination)	- Simple design and low cost - High selectivity (dependent on sequence design) - Signal amplification via nanomaterials
Aptamer-Based LFA Technology	High-affinity binding of aptamers to metal ions via conformational changes	- Recognizes multiple ions (Hg^2+^, Cd^2+^, etc.) - Stable aptamers with artificial screening capability - Supports multiplex detection
Nucleic Acid Enzyme-Based LFA Technology	Metal ions activate DNAzyme catalytic activity to cleave substrates and release signal molecules	- Catalytic amplification for ultrahigh sensitivity - Simple operation without complex instruments - Strong specificity for Pb^2+^, Cu^2+^, etc.
Antigen–Antibody-Based LFA Technology	Immunological binding of antibodies to metal ion–carrier protein complexes	- Mature commercialization with fast detection (3–4 min) - Compatible with fluorescence, SERS, and other signal modes - Suitable for regulatory standards

## Data Availability

Data are available upon request to the corresponding author.
